# Investigating the Scope of Resident Patient Care Handoffs within Neurosurgery

**DOI:** 10.1371/journal.pone.0041810

**Published:** 2012-07-27

**Authors:** Maya A. Babu, Brian V. Nahed, Robert F. Heary

**Affiliations:** 1 Department of Neurologic Surgery, Mayo Medical School, Rochester, Minnesota, United States of America; 2 Department of Neurosurgery, Massachusetts General Hospital, Boston, Massachusetts, United States of America; 3 Department of Neurosurgery, Neurological Institute of New Jersey, Newark, New Jersey, United States of America; University of New South Wales, Australia

## Abstract

**Introduction:**

Handoffs are defined as verbal and written communications during patient care transitions. With the passage of recent ACMGE work hour rules further limiting the hours interns can spend in the hospital, many fear that more handoffs will occur, putting patient safety at risk. The issue of handoffs has not been studied in the neurosurgical literature.

**Methods:**

A validated, 20-question online-survey was sent to neurosurgical residents in all 98 accredited U.S. neurosurgery programs. Survey results were analyzed using tabulations.

**Results:**

449 surveys were completed yielding a 56% response rate. 63% of neurosurgical residents surveyed had not received formal instruction in what constitutes an effective handoff; 24% believe there is high to moderate variability among their co-residents in terms of the quality of the handoff provided; 55% experience three or more interruptions during handoffs on average. 90% of neurosurgical residents surveyed say that handoff most often occurs in a quiet, private area and 56% report a high level of comfort for knowing the potential acute, critical issues affecting a patient when receiving a handoff.

**Conclusions:**

There needs to be more focused education devoted to learning effective patient-care handoffs in neurosurgical training programs. Increasingly, handing off a patient adequately and safely is becoming a required skill of residency.

## Introduction

Handoffs are defined as verbal and written communications occurring between healthcare professionals (attending physicians, resident physicians, and nurses) as they transition between work shifts [1]. Handoffs refer to the process of communication occurring when someone who has been caring for a patient transfers primary responsibility of that care to another person. For instance, a resident who has completed an on-call shift may handoff information on patients within their census to another resident who will be assuming care for those patients.

The Accreditation Council for Graduate Medical Education (ACGME) and the Institute of Medicine (IOM) recently released guidelines limiting the number of hours that can be worked by resident physicians to minimize fatigue [2], [3]. With mandated break periods in between patient care shifts, the need to “handoff,” or transfer patient care responsibilities between members of a healthcare team, has increased as housestaff physicians (residents and fellows) transition between shifts [4]. Duty-hour restrictions have led to an increased interest into investigating the safety and efficacy of these handoffs [5], [6].

The highly complex scientific fields of aviation, nuclear power, and complex DNA research have explored ways to train and ensure reliable handoffs [7]. Disciplines such as nursing [8], and some specialties within medicine, including Pediatrics [9], [10], Emergency Medicine [11], Obstetrics [12] and Internal Medicine [13], [14], [15], have studied handoffs, and have made recommendations as to which elements should be included in a proper and effective handoff. However, in the neurosurgical community, there has been no literature, to date, which addresses what constitutes an effective handoff and what elements of patient care need to be communicated in order to make an adequate and safe patient transfer.

Neurosurgery, apart from other surgical disciplines, has some distinct and unique challenges in managing patients. For instance, the management of external ventricular drain (EVD) settings in patients with elevated intracranial pressure (ICP) can impact patient outcomes and long-term treatment results if acute intervention is not promptly administered. The decision points in this scenario rest on an understanding of the patient's serial neurological examinations as well as trends in ICP measurements. Unique to the field of neurosurgery is the decision-making process as to when operative treatment needs to be instituted, when the EVD needs to be replaced, or when continued observation is acceptable. Each of these treatment paths can have direct positive or negative effects on the patient's ultimate neurological outcome.

The ability to accurately make decisions, based on serial observations with accurate handoff communications are specific to the field of neurosurgery. Neurosurgical programs are particularly vulnerable to the handoff situation by virtue of the comparatively smaller ratio of numbers of residents to the number of patients cared for. Most neurosurgical residency training programs employ between 1–2 residents per year. Typically, one or two neurosurgery residents are on-call per night covering a large patient census, managing numerous patients with acute neurosurgical issues, responding to Emergency Department consults, and possibly operating on urgent/emergent cases. It is unfeasible for the neurosurgical resident on-call to review the medical records of every patient he/she is covering. Thus, having concise but thorough patient handoffs is important and essential for patient safety. Currently the quality of handoffs depends largely on the individual neurosurgical resident to convey what he or she deems to be the most pertinent information. An improved system might include recommendations and best practices which are disseminated throughout the neurosurgical community. This paper explores the role of handoffs in facilitating patient safety, discusses the results of a survey of common practices among neurosurgical residents from across the country, and proposes several best practices for improving handoff communication.

### ACGME/IOM Guidelines

Reduced resident physician duty hours, first introduced in New York State in 1989 [16], were recommended for United States residency training programs in 1993, with six specialties (Emergency Medicine, Internal Medicine, Dermatology, Ophthalmology, Allergy/Immunology, and Preventive Medicine) adopting the newly proposed requirements [17]. In July, 2003, the ACGME instituted an 80-hour per week work restriction for residents across all residency programs [18]. In 2007, the Joint Commission on Accreditation of Healthcare Organizations (JCAHO) placed an emphasis on patient care handoffs when it required a standardized handoff process be implemented in order for a hospital to successfully receive accreditation [19]. On September 28, 2010, the ACGME Board of Directors approved new requirements for residency programs, including updated standards for resident duty hours, education, and supervision based upon specific recommendations from the Institute of Medicine (IOM): (1) the maximum number of work hours remains at 80 hours per week, averaged over 4 weeks; (2) moonlighting, both internal and external, is counted against the 80-hour weekly limit; and (3) duty periods are limited to 16 continuous hours (only applies to first-year residents). The ACGME recommended “strategic napping” after determining that the original IOM committee's recommendation of an uninterrupted 5-hour sleep period was “unworkable.” These new standards were implemented on July 1^st^, 2011 [20].

Prior work hour restrictions, which limited duty hours to 80 hours per week, have been studied in the literature. One survey queried the experiences of surgery program directors with the current ACGME duty-hour standards and asked for commentary views on the IOM's proposed duty-hour recommendations [21]. A total of 118 program directors (47.6% of all U.S. surgery programs) responded to the survey. The results showed that the current duty-hour standards have hindered clinical educational opportunities by reducing and/or eliminating: 1) rotations on many services; 2) didactic teaching conferences; and 3) clinical bedside teaching opportunities. Additionally, patient safety was perceived to have been compromised by frequent handoffs of care. With the exception of the moonlighting recommendation, most of the other IOM recommendations were perceived as “extremely difficult” or “impossible” to implement. These survey results strongly suggest that surgical program directors are very concerned about the newly determined duty-hours guidelines.

Given the variability across disciplines and operating room obligations often necessitating surgical residents work above 80 hours per week, the ACGME allowed residency programs to apply for a maximum of a 10% exemption to the 80 hours, extending the restriction to 88 hours per week. A certain proportion of neurosurgical residency programs have applied to the ACGME Neurosurgical Residency Review Committee for this 10% increase in maximum duty hours. There are 15 neurosurgical programs that have applied for and received the exemption; neurosurgery is the only field that has granted these exceptions; no other residency review committee has granted exceptions [22]. This suggests the ACGME acknowledges the unique demands of a neurosurgical service compared to other surgical and non-surgical residency training programs.

### Handoffs: Background

Handoffs occur during the transfer of a patient's location–for instance, between the Intensive Care Unit (ICU) and the step-down unit; between disciplines–for instance, Neurology to Neurosurgery; or within a discipline–for instance, by nurses, resident or attending physicians transferring care of patients at the change of shift. Transfers of information also occur throughout the care of a patient, beginning with the ambulance crew and the Emergency Department, the Emergency Department and the consultant, inpatient service, ICU, operating suites, and ultimately to out-of-hospital care providers. Each transfer of information has the potential for communication failure. As of 2006, 60% of training programs had no formal process for teaching interns how to transfer responsibility for patients to other physician caretakers [23]. Only 8% of U.S. medical schools formally taught handoff techniques in a lecture/small-group session as of 2005 [6]. As a result, resident physicians have been learned handoff techniques without formal training or evaluation of their performance.

According to JCAHO, inadequate communication between care providers or between care providers and patients/families is frequently associated with so-called “sentinel events,” or an unexpected occurrence involving death or serious physical or psychological injury. Communication failure was the primary root cause of 65% of reported sentinel events in 2006 [24]. One study at an urban tertiary care center studied the relationships between adverse events and handoffs of patient care. This study suggested that 44% of 124 consecutive adverse events reported were preventable. Patients with potentially preventable adverse events were more likely than control subjects to be covered by a physician from another team at the time of the event. Utilizing multivariate analysis, the presence of cross-coverage independently correlated with the presence of potentially preventable adverse outcomes (odds ratio, 6.1; 95% confidence interval, 1.4 to 26.7). The likelihood of occurrence of a preventable adverse event was also directly proportional to the severity of illness (Acute Physiology and Chronic Health Evaluation [APACHE] II score odds ratio per point, 1.2; 95% confidence interval, 1.1 to 1.4) [25].

Beyond adverse events, cross-coverage and gaps in communication can lead to ordering additional tests and procedures which drive up costs for patients and society. A study of the Minneapolis Veterans Affairs Medical Center examined the admission coverage system of the internal medicine residency program that had, on alternate days, assigned patients admitted the hospital either to the care of a senior resident providing continuous coverage within a team, or to a senior resident who transferred the patient's care to a different service on the following day [26]. Patients in the cross-cover group had significantly more tests performed and a significantly longer median length of hospital stay. When patients are transferred between facilities without complete handoffs and records in terms of documentation of tests, procedures, and interventions performed, there can be considerable duplication of studies which additionally drives up the cost of healthcare expenditures.

Another study of transfers between two centers found duplication of testing in 32% of cases; in 20% of these cases at least one duplicate test was not clinically indicated [27]. Unknown or unclear medication histories during patient handoffs can lead to potential medication adverse events [28].

There are medico-legal implications for improper handoffs, making it a multi-dimensional issue not limited to education and resident training [29]. An analysis of malpractice claims suggests that failures in handoff communications can be associated with a significantly increased risk of litigation. Among 240 cases of malpractice claims in which trainees were judged to have played an important role, teamwork breakdowns accounted for 167 of 240 contributing causes (70%), a larger frequency than that related to a lack of technical competence (139 of 240 causes; 58%) [30]. Lack of supervision and handoff problems were the most prevalent types of teamwork issues and both were disproportionately common among the errors that involved trainees.

Comparisons have been drawn between the rapid transfer of information between individuals in medicine and other fields, such as race-car driving. Three themes identified by examining pit stop crews in motor racing in the UK include: (1) proactive learning with briefings and checklists to prevent errors; (2) active management using technology to transfer information, and (3) post-hoc learning from the storage and analysis of electronic data records. Improvements in communication and capturing these through ongoing iterative improvement strategies are vitally important to any high stress industry [31].

## Methods

The neurosurgical literature lacks studies on the type of handoffs that prove to be an effective means for assessment. Instead, studies of *never* events, such as wrong-site craniotomies, have found that a failed communication between members of the healthcare team, while not classically fitting the definition of a patient handoff, bear culpability for wrong-site surgery [32], [33].

To study the issue of patient care handoffs among neurosurgical residents, we surveyed all residents in the 98 accredited neurosurgical training programs in the United States. We utilized an online web-based survey instrument to administer a short survey. We emailed the link for the survey to each neurosurgical resident in the United States.

There are approximately 795 residents in the country [34]; 449 completed our survey yielding a 56% survey response rate. We asked 18 questions about handoffs and 2 questions related to demographics (size of program and year in program) for a total of 20 questions. Prior to launch of the survey, we conducted a pilot during which we sampled all of the residents (14 residents) at one neurosurgical program (UMDNJ-NJMS) to determine (a) the time it takes to complete our survey questions (b) gauge ambiguity in the questions (c) receive feedback on the appropriate order of the questions. The UMDNJ-NJMS neurosurgical residents averaged 3 minutes to complete the survey. Using their feedback and insight, we revised the questions and produced the final online survey tool.

In most centers, attending neurosurgeons play a small role in supervising handoffs between residents. Instead, senior residents play more of a role in terms of providing feedback for the handoff process. Our survey sought to determine in part the role of senior residents in providing oversight during the handoff process. Specifically, our survey queried how often handoffs were monitored by a senior resident. Senior residents were also queried as to whether they received feedback from other senior residents as to the quality of their handoff. In some programs, evaluations are made by fellow residents, and our survey attempted to capture whether this feedback occurs in the context of patient handoffs.

## Results

The results are summarized in Tables 1, 2, 3, 4, 5, 6, 7, 8, 9, 10, 11, 12, 13, 14, 15, 16, 17, 18, 19, 20. Of note, we found that:

1. A significant number (63%) of neurosurgical residents surveyed have not received formal instruction in what constitutes an effective handoff;
2. 24% of neurosurgical residents surveyed believe there is high to moderate variability among residents in terms of the quality of the handoff provided;
3. 55% of neurosurgical residents surveyed experience three or more interruptions during handoff on average;
4. When receiving handoff, 10% of neurosurgical residents surveyed state that tasks requiring follow-up are not clearly identified at least some of the time; and
5. 47% of neurosurgical residents surveyed say that feedback on how they handoff is lacking.

**Table 1:** Survey Questions and Number of Responses, Question One.

(1) How much time on average do you take to sign-out from a neurosurgical colleague per 20 patients?							
Question	1 min or <	1–3 min	4–6 min	6–10 min	10–15 min	15–20 min	>20 min
Respondents	3	12	23	92	98	101	131
Percentage	1%	3%	5%	20%	21%	22%	28%

**Table 2:** Survey Questions and Number of Responses, Question Two.

(2) Do you sign-out in person or over the phone? (Check all that apply)						
Question	Always in person	Sometimes in person	Rarely in person	Always over the phone	Sometimes over the phone	Rarely over the phone
Respondents	269	146	5	3	128	76
Percentage	43%	23%	1%	.4%	20%	12%

**Table 3:** Survey Questions and Number of Responses, Question Three.

(3) Is the area quiet when you sign-out (i.e. can you hear all of the verbal communications of sign-out)? Check all that apply				
Question	Private room	Open nurses station	Cafeteria	Noisy area
Respondents	405	109	30	36
Percentage	70%	19%	5%	6%

**Table 4:** Survey Questions and Number of Responses, Question Four.

(4) Do you follow a standard protocol for sign-out?		
Question	Yes	No
Respondents	209	250
Percentage	46%	54%

**Table 5:** Survey Questions and Number of Responses, Question Five.

(5) If you have a protocol for handoffs, who designed the protocol?			
Question	Fellow residents	My residency program	Specialty society
Respondents	205	22	0
Percentage	90%	10%	0%

**Table 6:** Survey Questions and Number of Responses, Question Six.

(6) When you are the person receiving sign-out, how often do you ask questions?				
Question	Always	Sometimes	Rarely	Never
Respondents	327	125	6	1
Percentage	71%	27%	1%	.2%

**Table 7:** Survey Questions and Number of Responses, Question Seven.

(7) Is the sign-out you provide to others written, verbal, or both?			
Question	Written	Verbal	Both
Respondents	7	163	289
Percentage	2%	36%	63%

**Table 8:** Survey Questions and Number of Responses, Question Eight.

(8) How much background do you receive about the patient?			
Question	Only a one liner	A brief paragraph about the hospital course	Only communicate overnight/critical issues
Respondents	136	231	92
Percentage	30%	50%	20%

**Table 9:** Survey Questions and Number of Responses, Question Nine.

(9) How comfortable are you knowing the acute issues to watch for with each patient when you receive sign-out?					
Question	Always comfortable	Sometimes comfortable	Neutral	Rarely comfortable	Never comfortable
Respondents	252	174	28	4	0
Percentage	55%	38%	6%	1%	0%

**Table 10:** Survey Questions and Number of Responses, Question Ten.

(10) When you give sign-out, are you monitored by a senior resident?					
Question	Always	Usually	Sometimes	Rarely	Never
Respondents	84	96	90	82	100
Percentage	55%	38%	6%	18%	22%

**Table 11:** Survey Questions and Number of Responses, Question Eleven.

(11) Approximately how much time, on average, do you spend receiving sign-out for a new patient?					
Question	1 min or less	1–3 min	4–5 min	6–7 min	8–10 min
Respondents	29	240	126	30	23
Percentage	6%	54%	28%	7%	5%

**Table 12:** Survey Questions and Number of Responses, Question Twelve.

(12) Approximately how much time do you spend, on average, receiving sign-out on a patient known to you?					
Question	1 min or less	1–3 min	4–5 min	6–7 min	8–10 min
Respondents	279	148	17	3	2
Percentage	62%	33%	4%	1%	.4%

**Table 13:** Survey Questions and Number of Responses, Question Thirteen.

(13) When you receive sign-out, is it formatted by patient problem?					
Question	Always	Usually	Sometimes	Rarely	Never
Respondents	83	164	104	50	48
Percentage	18%	37%	23%	11%	11%

**Table 14:** Survey Questions and Number of Responses, Question Fourteen.

(14) When you receive sign-out, are the tasks you are supposed to do outlined for you?					
Question	Always	Usually	Sometimes	Rarely	Never
Respondents	255	151	31	6	6
Percentage	57%	34%	7%	1%	1%

**Table 15:** Survey Questions and Number of Responses, Question Fifteen.

(15) How many interruptions during sign-out (pager, etc) do you experience on average when you receive a sign-out?					
Question	Zero	1–2	3–4	5–6	7+
Respondents	25	175	146	48	55
Percentage	6%	39%	33%	11%	12%

**Table 16:** Survey Questions and Number of Responses, Question Sixteen.

(16) Do you find the ability to provide an adequate sign-out to be similar across residents in your program?					
Question	Highly similar	Somewhat similar	Neutral	Some variability	Highly variable
Respondents	111	187	39	76	34
Percentage	25%	42%	9%	17%	8%

**Table 17:** Survey Questions and Number of Responses, Question Seventeen.

(17) Have you ever received feedback on a sign-out you gave to another person?		
Question	Yes	No
Respondents	238	210
Percentage	53%	47%

**Table 18:** Survey Questions and Number of Responses, Question Eighteen.

(18) Have you ever formally received instruction as to what makes an adequate sign-out?		
Question	Yes	No
Respondents	165	284
Percentage	37%	63%

**Table 19:** Survey Questions and Number of Responses, Question Nineteen.

(19) How many residents are in your program?				
Question	0–7	8–14	15–21	21+
Respondents	62	152	103	14
Percentage	19%	46%	31%	4%

**Table 20:** Survey Questions and Number of Responses, Question Twenty.

(20) What is your year in your program?							
Question	PGY 1	PGY 2	PGY 3	PGY 4	PGY 5	PGY 6	PGY 7
Respondents	55	67	39	57	41	43	26
Percentage	17%	21%	12%	17%	13%	13%	8%

Despite these weaknesses apparent in current neurosurgical resident handoffs, there are several areas of strength:

1. A large proportion of neurosurgical residents surveyed (73%) report always asking questions when receiving a hand-off;
2. 90% of neurosurgical residents surveyed state that handoffs most often occurs in a quiet, private area;
3. 56% of neurosurgical residents surveyed report a high level of comfort for knowing the potential acute, critical issues affecting a patient when receiving handoffs.

We had respondents in programs of varying size, from less than seven residents to greater than 21 residents, and we also surveyed multiple years across residency, from PGY 1 to PGY 7 (Tables 1, 2, 3, 4, 5, 6, 7, 8, 9, 10, 11, 12, 13, 14, 15, 16, 17, 18, 19, 20). Not every question was answered by every respondent, as has been seen with similar multiple choice surveys published in the literature; however, the majority of neurosurgical residents answered all of the questions.

## Discussion

Given our survey findings and the best practices for handoff communications cited in the literature, we formulated several guiding principles for effective and safe patient handoffs in neurosurgery:

### A Model for Neurosurgery: Best Practices

#### (1) Tasks that require follow-up must be clearly identified

Critical issues that need to be followed up on (electrolyte concerns, hematological issues, coagulation pathway abnormalities, radiological imaging tests, excessive drain outputs or abnormal characterization of the draining fluid, or EEG findings) must be noted during handoffs in addition to the appropriate steps to follow depending on what the abnormality shows. If additional tests or interventions are necessary pending study results, this needs to be communicated to the on-call resident. Additionally, the time-sensitivity of the follow-up (immediate, urgent, semi-urgent) must be accurately communicated.

#### (2) More education on proper handoffs is necessary

While it is difficult to teach appropriate handoffs through a lecture-based format, not to mention that the environment of each program differs, residency programs should strive to improve handoffs with an ongoing, iterative learning process that is tailored for each program. Regular feedback on the handoff process should also be given to the residents.

#### (3) Interruptions must be kept to a minimum

Just as nurses have “protected” handoffs time, neurosurgical residents should also be protected from page interruptions during handoffs. The best method involves another neurosurgical resident covering the on-call pager while the handoffs is taking place. After the handoffs has been completed, the on-call resident takes the pager and begins his or her responsibilities. On weekends and holidays, this may not be possible as the “on-duty” resident may be turning over directly to the next resident coming “on duty” and the ability to have protected handoffs time may not be realistic. During these weekend/holiday times, emergent issues which warrant immediate attention would need to be attended to; however, general floor questions should not interrupt the handoffs. The idea of having a protected handoffs period must be communicated to the nursing staff and administration so that non-urgent pages during this time period are kept to a minimum and made with the understanding that the on-call resident will respond at the completion of the formal handoffs.

#### (4) Neurosurgical management issues must be highlighted during the handoffs

Patient management issues, including external ventricular drain settings, intracranial pressure monitors, seizure prophylaxis, etc., must be identified clearly, including the parameters warranting treatment and the type of intervention recommended. Baseline physical exam findings need to be communicated (egs. hemiparesis, facial droop, etc) such that if called overnight about a “change” in clinical exam, the on-call resident has a benchmark from which to make an assessment.

### Hand-off Research from Other Specialties

The literature is rich with studies of the safety and efficacy of handoffs in medicine, including peri-operative nursing and among medical interns [35], [36], [37], [38]. However, little has been written on the particular issues in neurosurgical handoffs, the generalizability of handoffs across institutions, how to engage in handoffs, and quality assurance in handoffs.

In the nursing literature, a study of 10 patients in Australia found that involving patients in shift-to-shift bedside nursing handoffs improved patients' perceptions of being engaged in handoffs and feeling that they had access to maintaining the accuracy of the information transferred [39]. A systematic review of the nursing literature on handoffs found that while many of the flaws of failed patient handoffs have been explored, there has been little identification of the best practices in developing effective patient handoffs [40], [41].

Internal medicine, in particular, has studied the impact of patient handoffs between interns. A study of 25 internal medicine interns at a single institution evaluated a curriculum developed to enhance the accuracy and reliability of handoffs and found persistent improvement in the completeness of handoffs sheets and the accuracy of patient reporting particulars two-months after the initiation of the curriculum. While this study only evaluated performance for two months from initial implementation, it suggests that use of a formal curriculum may provide structure and standardization which affects, and potentially improves, trainees' practices [13]. Another study evaluating 34 interns on a general internal medicine ward found that initiation of a standardized form significantly reduced the likelihood that the interns covering the night shift would miss handoffs data including important disease conditions, contingency plans, or medications (p<.003) [42]. Interestingly, standardized handoffs did not significantly alter the frequency of dropped tasks or missed laboratory and radiographic data as perceived by the night intern. However, the covering day residents thought there were significantly fewer perceived errors on the part of the night intern after introduction of the standardized handoffs sheet (p = .001).

Handoffs are directly related to the involved residents' clinical skills. This suggests a need for education and initial supervision of junior residents. Studies are needed on how to best conduct effective handoffs under shortened duty periods [43]. This should assess ways to transfer strategies and data summaries may enhance efficiency and effectiveness, and how these may substitute when a verbal interactive handoff is not feasible. Developing handoff skills should start as early as during medical school. In an evaluation of 60 medical students, Chu, et. al., found that developing handoff skills increased understanding and confidence in accepting patient handoffs [44]. There is further evidence to suggest that structured handoffs can affect patient safety [45] by influencing the knowledge and confidence of the accepting intern [46]. Other studies have questioned these findings, suggesting that handoffs may lead to incorrectly inflated perceptions and confidence in accepting patient care [47].

Personal surveys face inherent limitations. In terms of the present study, neurosurgery residents will likely feel obligated to some degree to voice negative opinions on work hours restrictions, which is indirectly queried by our assessment of handoffs. Therefore, one must remain mindful of the responses rendered in context of a surgical field's biases on the related topic of work hour restrictions.

Further study is needed to examine how the number of neurosurgical handoffs can influence medical errors especially given that neurosurgical handoffs tend to be more focused on neurosurgical issues, whereas other fields, such as Internal Medicine or Pediatrics, may have multi-systemic patient care issues that require handoffs.

Another shortcoming of our survey was that we failed to query how many handoffs occur over the course of a typical day or week. How often handoffs occur is highly variable across programs. In some programs, residents carry their service pagers throughout the week while at other programs, a resident covers the floor during the day while more senior residents operate. A night resident typically covers the service pager overnight. Understanding the type of handoff environment would have enhanced the ability to interpret our findings in the most appropriate context.

### Conclusion

As duty hour restrictions become more restrictive, housestaff are increasingly under pressure to perform more efficiently [25], [48], [49]. It is essential that systems be developed which permit the neurosurgical resident physicians to effectively utilize their time to accurately communicate patient care information between each other in a manner that optimizes patient outcomes and patient safety. Other fields have shown the benefits of more structured handoffs (both in medicine and other non-medical fields). The survey presented in this paper demonstrates a high degree of variability in the handoff procedures and flaws in the existing neurosurgical handoffs system occurring nationwide in neurosurgical residency training programs. When similar best practices of high-reliability organizations have been adopted by programs, performance metrics have improved [7], [31], [50]. As work-hour restrictions mandate more handoffs being required among neurosurgeons in the foreseeable future, it is incumbent upon the field of neurosurgery to develop the necessary procedures to improve patient care and patient safety.
